# Liquid Crystalline Assembly of Coil-Rod-Coil Molecules with Lateral Methyl Groups into 3-D Hexagonal and Tetragonal Assemblies

**DOI:** 10.3390/ijms15045634

**Published:** 2014-04-02

**Authors:** Zhuoshi Wang, Yu Lan, Keli Zhong, Yongri Liang, Tie Chen, Long Yi Jin

**Affiliations:** 1 Key Laboratory for Organism Resources of the Changbai Mountain and Functional Molecules, Department of Chemistry, College of Science, Yanbian University, No.977 Gongyuan Road, Yanji 133002, China; E-Mails: 2013010407@ybu.edu.cn (Z.W.); 2011010505@ybu.edu.cn (Y.L.); 2 College of Chemistry, Chemical Engineering and Food Safety, Bohai University, Jinzhou 121013, China; E-Mail: zhongkeli2000@bhu.edu.cn; 3 Beijing National Laboratory for Molecular Sciences, Joint Laboratory of Polymer Science and Materials, Institute of Chemistry, Chinese Academy of Sciences, Beijing 100190, China; E-Mail: liangyr@iccas.ac.cn

**Keywords:** coil-rod-coil, self-assembly, columnar, hexagonal, tetragonal, small angle X-ray scattering (SAXS)

## Abstract

In this paper, we report the synthesis and self-assembly behavior of coil-rod-coil molecules, consisting of three biphenyls linked through a vinylene unit as a conjugated rod segment and poly(ethylene oxide) (PEO) with a degree of polymerization (DP) of 7, 12 and 17, incorporating lateral methyl groups between the rod and coil segments as the coil segment. Self-organized investigation of these molecules by means of differential scanning calorimetry (DSC), thermal polarized optical microscopy (POM) and X-ray diffraction (XRD) reveals that the lateral methyl groups attached to the surface of rod and coil segments, dramatically influence the self-assembling behavior in the liquid-crystalline mesophase. Molecule **1** with a relatively short PEO coil length (DP = 7) self-assembles into rectangular and oblique 2-dimensional columnar assemblies, whereas molecules **2** and **3** with DP of 12 and 17 respectively, spontaneously self-organize into unusual 3-dimensional hexagonal close-packed or body-centered tetragonal assemblies.

## 1. Introduction

Well-ordered supramolecular materials with nanometer-scale architectures have shown a variety of photo-electro or bioactive functionalities in biochemistry, molecular electronics, and materials science [1–10]. Supramolecular nanostructures of organic or inorganic-organic hybrid materials can be created by self-assembly of these materials via weak intermolecular forces, such as non-covalent forces including hydrophobic and hydrophilic effects, electrostatic interaction, hydrogen bonding, and microphase segregation [11–15].

Self-assembling organic molecules, especially, the construction of supramolecular nanostructures with well defined shape and size from conjugated rod-coil molecular architecture is one of the great interesting issues in organic and bioorganic materials chemistry due to the fact that ordered and conjugated rod building blocks can exhibit unique electronic and photonic properties for application in nanomaterials science [16–20]. It has been reported that the supramolecular assemblies of coil-rod-coil molecules can be precisely tuned to form various nanostructure by the cooperative effects of several molecular parameters such as volume fraction of rod-to-coil segment, the cross-sectional area of coil segment, the shape of rigid rod segment and rod anisotropy [21–23]. For the past ten years, Lee, Percec and other groups reported the formation of 1-D lamellar, 2-D columnar and 3-D discrete bundles supramolecular nanostructures from the self-assembly of a series of rod-coil molecules consisting of three biphenyls connected through vinylene linkages as a conjugated rod segment, and poly(propylene oxide) (PPO), poly(ethylene oxide) (PEO) or aliphatic polyether dendritic wedges with different cross-sections as the coil segments [24–26]. For example, Lee reported a wedge-coil block conjugated rod-coil molecules consisting of a rigid wedge and a flexible poly(ethylene oxide) coils with DP (degree of polymerization) of 12, 21, 35 and 45 [27]. The authors demonstrated that the molecules self-assembled into 3D cubic, 2D hexagonal columnar, 3D perforated lamellar, and smectic-like lamellar structures in the liquid crystalline phase, as the length of the PEO chain increased. They also interpreted that compared to other self-assembling rod-coil architecture, the existence of an unusual phase transformation based on dendritic molecules from spherical cubic and columnar structures, into an unusual bilayer structure, was caused by the combination effect of shape complementarity and microphase separation between the conjugated rod and flexible PEO coil segments.

**Scheme 1.**
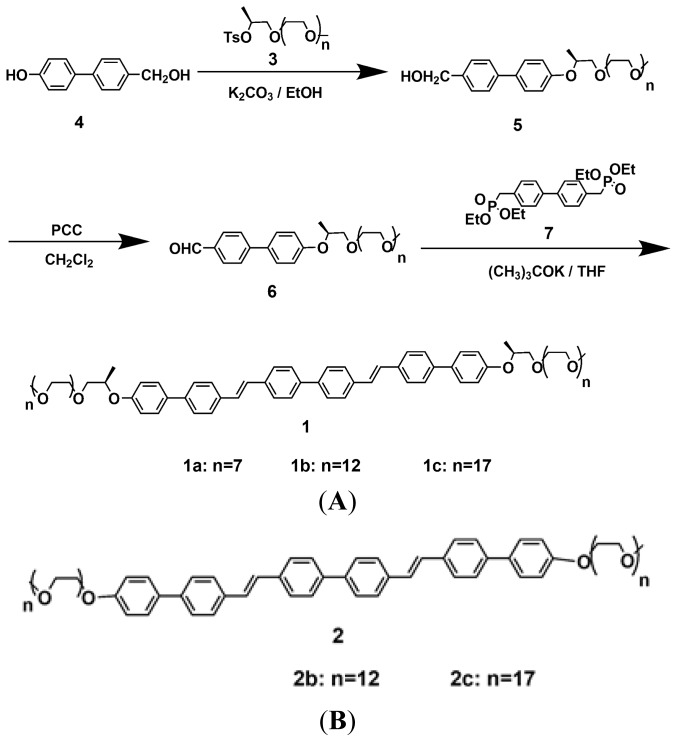
Synthetic route of coil-rod-coil molecules **1a**–**1c** (**A**) and chemical structures of molecules **2b** and **2c** (**B**).

In addition, in our previous work, we have reported the self-assembly of coil-rod-coil triblock molecules consisting of three biphenyls connected through vinylene linkages as a conjugated rod segment and poly(ethylene oxide) with a degree of polymerization of 12 and 17 as the coil segment (molecules **2b** and **2c**, see Scheme 1) [28]. The results of structural analysis indicate that these molecules spontaneously self-organize into a 3-D hexagonally perforated layer (HPL) structure and a 2-D rectangular or an oblique columnar structure in the crystalline and liquid crystalline phases, respectively.

To further investigate the influence of methyl groups at the surface of rod and coil segments for construction ordered supramolecular nanostructures [15,28,29], in this study, we designed and synthesized coil-rod-coil molecules consisting of three biphenyls linked through a vinylene unit as a conjugated rod segment and poly(ethylene oxide) with a degree of polymerization of 7, 12 and 17, incorporating lateral methyl groups between the rod and coil segment as the coil segment. The self-assembling behaviors of **1a**–**1c** was investigated by means of differential scanning calorimetry (DSC), thermal polarized optical microscopy (POM) and X-ray scattering (XRD).

## 2. Results and Discussion

### 2.1. Synthesis of Oligomers **1a**–**1c**

The synthetic route of coil-rod-coil molecules **1a**–**1c** consisting of three biphenyls connected through vinylene linkages as a conjugated rod segment and poly(ethylene oxide) incorporating lateral methyl groups between the rod and coil segment as the coil segment is described in Scheme 1. Molecules **1a**–**1c** successfully synthesized through the Wittg-Horner reaction of 4,4′-biphenylmethylbis( diethylphosphonate) (molecule **7**) and molecule **6** in the presence of potassium tertbutoxide. Molecules **6** with an aldehyde functional group were prepared from the oxidation reaction of **5** (see Scheme 1 and Figure S1) using pyridinium chlorochromate (PCC) as the oxidation reagent, in the methylene chloride solution at room temperature.

The structures of these molecules were characterized by ^1^H-NMR spectroscopy and a representative analysis of **1a** is described below. The presence of signals of aromatic protons at 7.64–7.53 ppm (m, 20H, o to CHCHphenyl, m to CHCHphenyl, o to phenylOCH_2_CH, m to phenylOCH_2_CH, m to OCH_2_CH_2_), 7.19 (s, 4H, CHphenyl), 7.02–7.00 (d, *J* = 8.3 Hz, 4H, o to phenylOCH_2_), of methylene protons linkaged with phenyl and ether bonds at 4.67–4.56 (m, 2H, CHCH_3_), 3.75–3.53 (m, 54H, OCH_2_CH_2_O), of metoxy protons at 3.38 (s, 6H, OCH_2_CH_2_OCH_3_), and of lateral methyl protons at 1.36–1.34 (d, *J* = 6.1 Hz, 6H, CHCH_3_) respectively, indicated that molecule **1a** was successfully synthesized (Figure S2). The MALDI-TOF mass spectrum of the molecule **1a** exhibit exact signals that can be assigned as the H^+^ labeled molecular ions; the experimental mass based on peak positions in the spectrum is well matched with the theoretical molecular weight of molecule **1a** (Figure S3). The structures of molecules **1b** and **1c** were also characterized by ^1^H-NMR spectroscopy and MALDI-TOF mass spectroscopy and were shown to be in full agreement with the structure presented in Scheme 1 (see Figures S4 and S5).

### 2.2. Self-Assembly of Molecules **1a**–**1c** in the Bulk State

The self-assembling behavior of the coil-rod-coil molecule **1** was investigated by means of differential scanning calorimetry (DSC), polarized optical microscopy (POM) and small-angle X-ray scatterings (SAXS). Figure S6 (see Supporting Information) shows the DSC heating, cooling traces and thermal transitions of the oligomers **1a**–**1c**. The transition temperatures together with the corresponding enthalpy changes (in brackets) determined from the DSC scans are summarized in Table 1. As shown in Table 1, the melting transition temperatures of the coil-rod-coil molecules decrease as the PEO coil length increase, especially, phase transition temperatures of molecules **1a**–**1c**, incorporating lateral methyl groups between the rod and coil segment, significantly decrease than molecules **2b**–**2c** (see Table S1) [28]. The results clearly imply that increasing flexible chain length or incorporating side groups at the surface of rod and coil domains leads to reduced phase transition temperatures [30,31].

**Figure 1.**
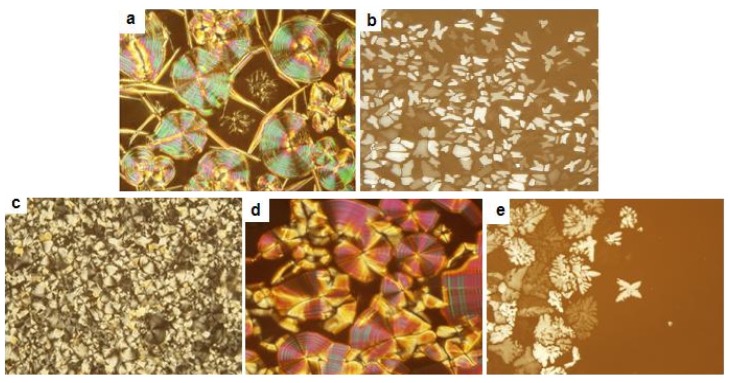
Representative optical polarized micrograph (×100) of the texture exhibited by (**a**) hexagonal perforated lamellar structure of **1a** in the crystal phase; (**b**) Rectangular columnar structure of **1a** and (**c**) oblique columnar structure of **1a** at the transition from the anisotropic liquid crystal phase; (**d**) 3-D hexagonal close-packed structure of **1b** at the transition from the anisotropic liquid crystalline phase; (**e**) Body-centered tetragonal micelle of **1c** at the transition from the anisotropic liquid crystalline phase.

**Figure 2.**
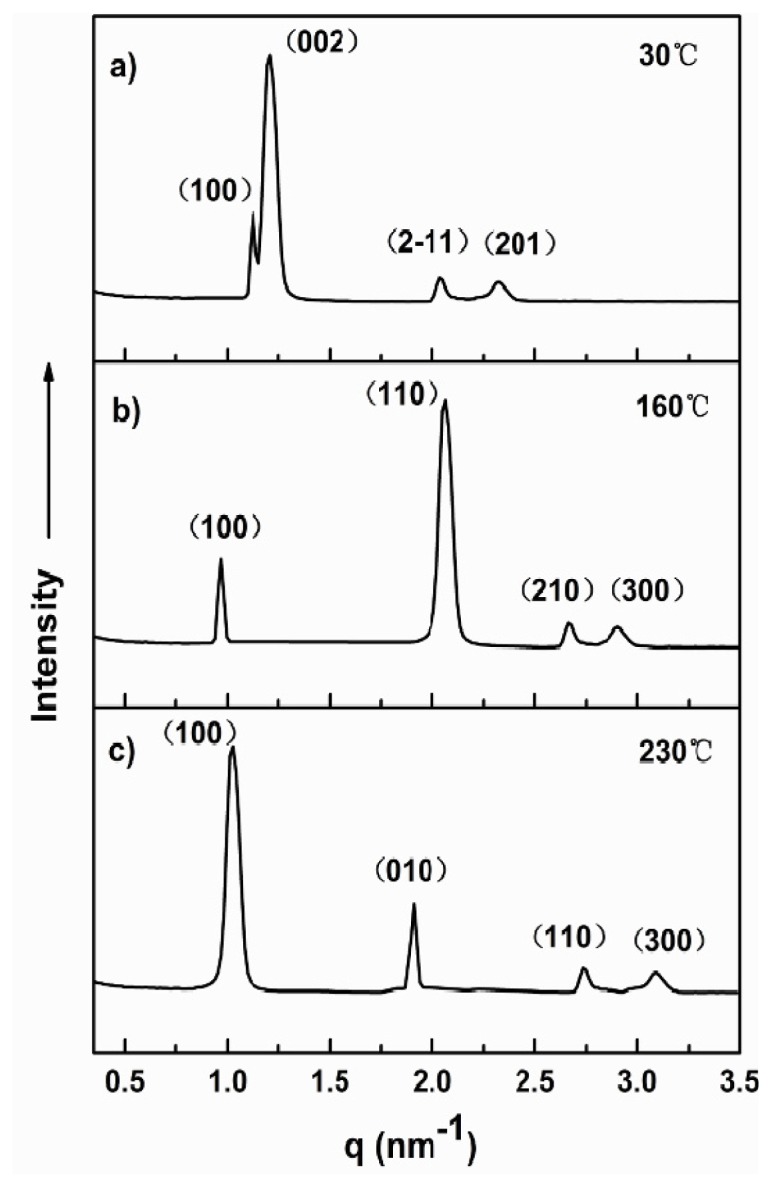
Small-angle and wide-angle X-ray diffraction patterns of **1a** plotted against *q* (=4πsin2θ/λ). (**a**) Hexagonal perforated layer at 30 °C; (**b**) Rectangular column at 160 °C; and (**c**) Oblique column at 230 °C.

On slow cooling from the isotropic state of **1a**, a pseudo focal conic with arched striation texture was observed on optical polarized microscope, indicating the presence of hexagonally ordered crystalline phase (Figure 1a). To identify the detailed self-assembled nanostructures, the small-angle X-ray scattering experiments of molecules **1a**–**1c** were performed in their solid and melt states. Figure 2 presents SAXS diffraction patterns for molecule **1a** measured at different temperatures. In the crystalline phases, the SAXS patterns display two main peaks together with several reflections with low intensity, as shown in Figure 2a. The observed reflections can be indexed as the (100), (002), (2–11) and (201) planes for 3-D hexagonal symmetry (P63/mmc space group symmetry) with lattice constants *a* = 6.48 nm and *c* = 10.38 nm (Table 2). An interesting point to be noted here is that the peak intensity associated with the (002) reflection appears to be the most intense, implying that the fundamental structure is lamellar. Hence, the supramolecular structure of **1a** in the crystalline phases can be characterized as a honeycomb like crystalline layer of the rod segments with in-plane hexagonal packing of coil perforations. From the lattice parameters determined from the X-ray diffraction patterns and the densities of each segment, the perforation sizes in diameter are calculated to be 7.94 nm. Upon cooling from the isotropic liquid, a leaf-like texture with the characteristic of a columnar mesophase can be observed at 160 °C by using an optical polarized microscope (Figure 1b). The detailed liquid crystalline structure is also confirmed by small-angle X-ray scattering experiments.

The SAXS diffraction pattern shows one strong reflection, together with three reflections with low intensity at higher angles (Figure 2b). These reflections can be assigned as the (100), (110), (210) and (300) planes for 2-D rectangular symmetry with lattice parameters of *a* = 6.49 nm and *b* = 3.43 nm (Table 2). From the experimental values of the unit cell parameters (*a*, *b*, *c*) and the density (*ρ* = 1.02), the average number (*n*) of molecules per cross-sectional slice of the column is calculated as about four, according to following equation [31], where *M_W_* is the molecular weight and *N_A_* is Avogadro’s number.

(1) $$n=(abc\times \text{sin}\gamma )\times \rho \times N_{A}/M_{W}$$

As shown in Figure 1c, the POM pattern measured at 230 °C shows focal conical spherulitic fan texture, also indicating the presence of columnar ordered liquid crystalline phase (Figure 1c). The SAXS scattering of **1a** recorded at 230 °C displays one sharp reflection together with three reflections that can be indexed as the (100), (010), (110) and (300) that correspond to a two-dimensional oblique columnar with a characteristic angle γ = 135 of oblique columnar and lattice parameters *a* = 8.61 nm and *b* = 4.66 nm (Figure 2c and Table 2). The average number (*n*) of molecules per cross-sectional slice of the column is calculated as about six, according to Equation (1).

**Figure 3.**
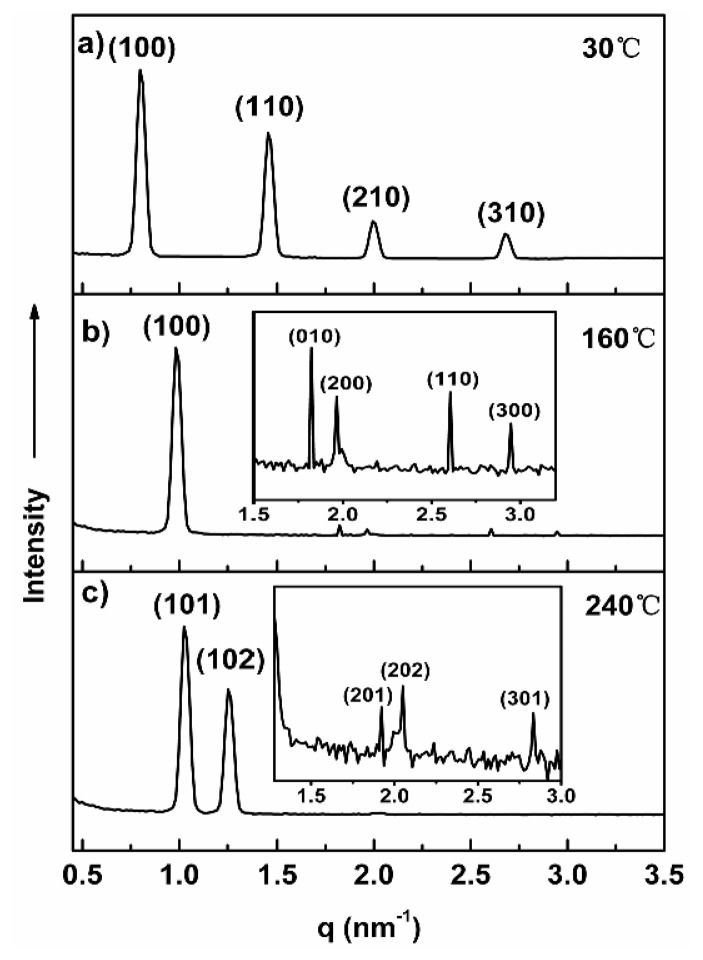
Small-angle and wide-angle X-ray diffraction patterns of **1b** plotted against *q* (=4πsin2θ/λ). (**a**) Rectangular column at 30 °C; (**b**) Oblique column at 160 °C; and (**c**) Hexagonal close-packed structure measured at 240 °C.

To investigate the cooperative effect between the methyl groups at the surface of rod and coil segments, and coil length for the creation of supramolecular nanostructures, we synthesized molecule **1b** and **1c** with longer PEO coil chains than molecule **1a**. For molecule **1b**, X-ray diffraction patterns shown in Figure 3a can be indexed as the (100), (110), (210) and (310) planes that correspond to a two-dimensional rectangular columnar with lattice constants *a* = 7.9 nm, *b* = 5.2 nm (Table 3) [32,33]. From the experimental values of the unit cell parameters (*a*, *b*, *c*) and the density (*ρ* = 1.02), the average number (*n*) of molecules per cross-sectional slice of the column is calculated as about 8, according to Equation (1). Upon heating the sample, the SAXS diffraction pattern of molecule **1b** in the melt state recorded at 160 °C shows one strong reflection, together with three reflections with low intensity at higher angles (Figure 3b), which can be assigned as the (100), (010), (200), (110) and (300) planes for 2-D oblique columnar with lattice parameters of *a* = 9.04 nm and *b* = 4.89 nm, characteristic angle γ = 135 (Table 3).

The average number (*n*) of molecules per cross-sectional slice of the column is calculated as about seven. In sharp contrast to the lower temperature liquid-crystalline phase, a pseudo focal conic with arched striation texture was observed on optical polarized microscope (Figure 1d), and the small-angle X-ray diffraction pattern of **1b** measured at 240 °C shown in Figure 3c display two well-resolved reflections with several low intensity peaks, which can be assigned as the (101), (102), (201), (202) and (301) planes for a 3-D hexagonal order (P63/mmc space group symmetry) with lattice constants *a* = 7.75 nm and *c* = 15.2 nm (Table 3). The peak intensities indexed as 101 and 102 reflections appeared to be relatively strong, as opposed to a hexagonally perforated lamellar structure where the peak intensity associated with the (002) reflection appears to be the most intense, implying that the fundamental structure of **1b** in the liquid crystalline is not lamellar lattice. The results of small angle X-ray scattering analysis, together with optical microscopic observations, confirm that the fundamental structure of the 3-D hexagonal structure is based on discrete bundles. In this 3-D hexagonal order system, molecule **1b** self-assembles into discrete rod-bundles that are encapsulated by PEO coils and subsequently organize into a 3-dimensional hexagonal close-packed structure. To describe the detailed supramolecular nanostructure, it is desirable to calculate the number of molecules per micelle. The number of molecules in a single rod bundle can be calculated as about 145, according to Equation (2), where *a*, *c* and *ρ* are the lattice constants and the density of molecule.

(2) $$n=a^{2}c\frac{N_{A}\rho }{2M_{W}}$$

**Figure 4.**
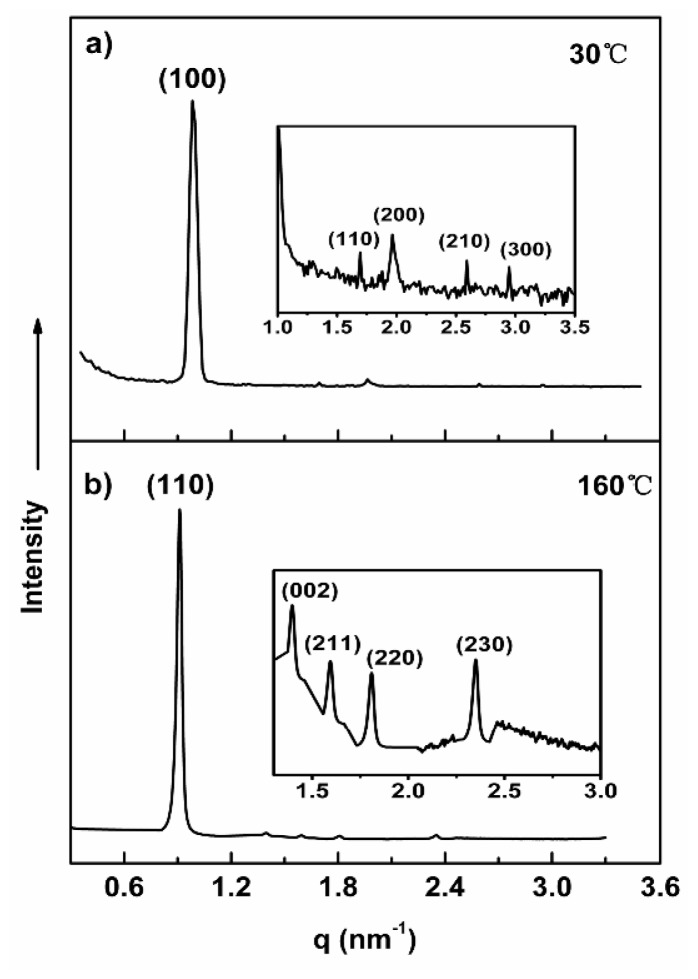
Small-angle and wide-angle X-ray diffraction patterns of **1c** plotted against *q* (=4πsin2θ/λ). (**a**) Hexagonal column at 30 °C; (**b**) Body-centered tetragonal structure at 160 °C.

For molecule **1c**, we also performed SAXS and POM experiments for evaluating the self-assembling nanostructures in the bulk state. As shown in Figure 4a, in the lower temperature crystalline phase, several diffraction peaks with the ratio of 1:√3:√4:√7:√9 in the small-angle region can be indexed as the (100), (110), (200), (210), (300) reflections of a 2D hexagonal columnar structure (Table 4 and Figure S7). From the observed d spacing of the 100 reflection, the lattice parameter of 2D hexagonal columnar phase of **1c** is calculated to be 7.4 nm, and the average number (*n*) of molecules per cross-sectional slice of the column is calculated as about seven, according to Equation (1). In the liquid crystalline phase of **1c**, the POM texture of **1c** observed at 160 °C shows fern-like domains with rectangular shape growing in four directions (Figure S7). Meanwhile the small-angle X-ray diffraction pattern shows a sharp, high intensity reflection at a low angle together with a number of sharp reflections of low intensity at higher angles (Figure 4b). These reflections can be indexed as the (110), (002), (211), (220), (230) planes for a three-dimensional body-centered tetragonal structure with lattice constants of *a* = 9.8 nm and *c* = 9.02 nm (*c*/*a* = 0.92) (Table 4) [29]. The average number (*n*) of molecules per micelle can be calculated as about 132, according to Equation (2).

**Figure 5.**
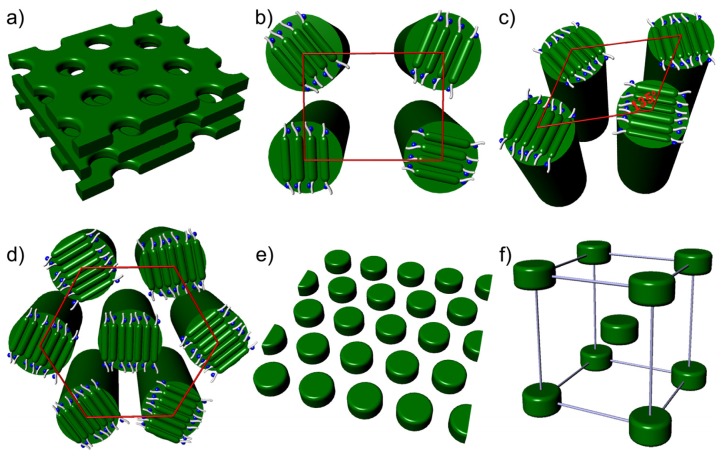
Schematic representation of self-assembly of (**a**) hexagonal perforated layer structure for **1a**; (**b**) Rectangular columnar structure for **1a**; (**c**) Oblique columnar structure for **1a**; (**d**) Hexagonal column for **1c**; (**e**) Hexagonal close-packed structure for **1b**; (**f**) Body-centered tetragonal structure for **1c**.

On the basis of the above discussion, we draw the interesting conclusion that molecules **1a** self-assemble into hexagonal perforated lamellar, rectangular columnar and oblique columnar nanostructures in the solid state and the liquid crystalline phase. However, molecules **1b** and **1c** with a longer PEO coil chains than **1a**, self-organize into columnar and hexagonal or tetragonal 3-D nanostructures in the solid state and the liquid crystalline phase. The variation of supramolecular nanostructures constructed by these molecules can be rationalized by considering the microphase separation between the dissimilar parts of the molecules and the space-filling requirements of the flexible PEO chains. Based upon the data presented so far, a schematic representation of the self-assembled structures of **1a**–**1c** is illustrated in Figure 5.

It should be pointed out that as mentioned in the introduction, in contrast with molecules **1a**–**1c**, molecules **2b** and **2c** self-assemble into perforated lamellar structures and columnar assemblies in the crystalline phase and in the liquid crystalline mesophase. Hence, it is of great interest that incorporating methyl groups at the surface of the rod and coil segments can create supramolecular structure: from the 2-dimensional columnar assemblies to a 3-dimensional hexagonal close-packed or body-centered tetragonal assemblies of molecules **1b** and **1c** in the liquid-crystalline mesophase. Therefore, the lateral methyl group at the surface of rod and coil segments is one of the main parameters for influencing the self-assembling behavior of the coil-rod-coil molecular system. The methyl groups at the surface of rod and coil segments can lead to decreased interaction of π-π stacking, and subsequently loosen the packing of the rod segments to produce more stable 3D bundle nanostructures. Thus, the strategy of incorporating lateral methyl groups into the interface coil-rod-coil molecular architecture can produce three dimensional supramolecular nonostructures, as well as adjusting coil volume fraction or chain length of rod-coil molecular system.

## 3. Experimental Section

### 3.1. Materials

4′-Hydroxy-4-biphenyl methylene alcohol (99%), pyridinium chlorochromate (PCC, 98%), potassium tertbutoxide (97%), toluene-*p*-sulfonyl chloride (99%), (−)-Ethyl l-lactate, 3,4-Dihydro-2H-pyran, 3,4-Dihydro-2H-pyran (99%), poly (ethylene oxide) monomethyl ether of DP 7, 12 and 17 (all from J&K CHEMICAL LTD, Shanghai, China, and Sigma-Aldrich China, Inc., Shanghai, China) and the other conventional reagents were used as received. Compounds **3**–**5** and phosphonium salt **7** (see Supplementary Information) was synthesized according to the procedure described elsewhere [31,33,34].

### 3.2. Techniques

^1^H-NMR spectra was recorded from CDCl_3_ solution on a Bruker AM 300 spectrometer. Column chromatography (silica gel 100–200) was used to check the purity of the products. A Perkin Elmer Pyris Diamond differential scanning calorimeter (Perkin Elmer, Waltham, MA, USA) was used to determine the thermal transitions with the maxima and minima of their endothermic or exothermic peaks, controlling the heating and cooling rates to 10 °C/min. X-ray scattering measurements were performed in transmission mode with synchrotron radiation at the 1W2A X-ray beam line at Beijing Accelerator Laboratory (Beijing, China). MALDI-TOF-MS was performed on a Perceptive Biosystems Voyager-DE STR (Applied Biosystems, Foster City, CA, USA) using a 2-cyano-3-(4-hydroxyphenyl) acrylic acid (CHCA) as matrix.

### 3.3. Synthesis Compounds **6a**–**6c**

Compounds **6a**–**6c** were all synthesized using the same procedure. A representative example is described for **6a**. Compound **5a** (1 g, 1.97 mmol) was dissolved in 50 mL of methylene chloride. PCC (1.15 g, 5.36 mmol) was added under nitrogen. The reaction mixture was stirred at room temperature under nitrogen for 6 h. The resulting solution was removed in a rotary evaporator, and the crude product was purified by column chromatography (silica gel, ethyl ether eluent) to yield 0.75 g (75%). ^1^H-NMR (300 MHz, CDCl_3_, δ, ppm): 10.04 (s, 1H, phenyl*CHO*), 7.94–7.91 (d, *J* = 8.0 Hz, 2H, *o* to *phenyl*CHO), 7.73–7.70 (d, *J* = 7.9 Hz, 2H, *m* to *phenyl*CHO), 7.59–7.56 (d, *J* = 8.2 Hz, 2Ar-H, *o* to *phenyl*CHO), 7.04–7.02 (d, *J* = 8.3 Hz, 2H, *m* to *phenyl*CHO), 4.71–4.56 (m, 1H, *CH*CH_3_), 3.80–3.55 (m, 36H, O*CH_2_CH_2_*), 3.38 (s, 3H, OCH_2_CH_2_*OCH_3_*), 1.36–1.34 (d, *J* = 5.7 Hz, 6H, CHC*H*_3_).

Compound **6b**: yield: 77%; ^1^H-NMR (300 MHz, CDCl_3_, δ, ppm): 10.04(s, 1H, phenyl*CHO*), 7.94–7.91 (d, *J* = 7.7 Hz, 2H, *o* to *phenyl*CHO), 7.73–7.70 (d, *J* = 7.7 Hz, 2H, *m* to *phenyl*CHO), 7.59–7.56 (d, *J* = 7.1 Hz, 2Ar-H, *o* to *phenyl*CHO), 7.04–7.02 (d, *J* = 7.4 Hz, 2H, *m* to *phenyl*CHO), 4.69–4.59 (m, 1H, *CH*CH_3_), 3.80–3.55 (m, 55H, O*CH_2_CH_2_*), 3.38 (s, 3H, OCH_2_CH_2_*OCH_3_*), 1.38–1.33 (d, *J* = 5.7 Hz, 6H, CHC*H*_3_).

Compound **6c**: yield: 78%; ^1^H-NMR (300 MHz, CDCl_3_, δ, ppm): 10.04(s, 1H, phenyl*CHO*), 7.94–7.91 (d, *J* = 7.6 Hz, 2H, *o* to *phenyl*CHO), 7.73–7.70 (d, *J* = 7.7 Hz, 2H, *m* to *phenyl*CHO), 7.59–7.56 (d, *J* = 7.1 Hz, 2Ar-H, *o* to *phenyl*CHO), 7.04–7.02 (d, *J* = 7.4 Hz, 2H, *m* to *phenyl*CHO), 4.70–4.60 (m, 1H, *CH*CH_3_), 3.79–3.54 (m, 69H, O*CH_2_CH_2_*), 3.38 (s, 3H, OCH_2_CH_2_*OCH_3_*), 1.37–1.33 (d, *J* = 5.6 Hz, 6H, CHC*H*_3_).

### 3.4. Synthesis Oligomers **1a**–**1c**

Oligomers **1a**–**1c** were all synthesized using the same procedure. A representative example is described for **8a**. Compound **6a** (0.75 g, 1.34 mmol) was dissolved in 30 mL dry THF. A solution of phosphonium salt (0.12 g, 0.27 mmol) and KOC(CH_3_)_3_ (0.226 g, 2 mmol) in 30 mL dry THF was added under nitrogen. The reaction mixture was stirred at room temperature under nitrogen for 24 h. The resulting solution was removed in a rotary evaporator. The crude product was extracted with methylene chloride and water. The methylene chloride solution was washed with water, dried over anhydrous magnesium sulfate, and filtered. The solvent was removed in a rotary evaporator, and the crude product was purified by column chromatography (silica gel, ethyl acetate eluent) to yield 0.15 g (42%) of a yellow green solid.

Oligomer **1a**: yield: 42%; ^1^H-NMR (300 MHz, CDCl_3_, δ, ppm): 7.64–7.53 (m, 20H, *o* to CHCH*phenyl*, *m* to CHCH*phenyl*, *o* to *phenyl*OCH_2_CH, *m* to *phenyl*OCH_2_CH, *m* to OCH_2_CH_2_), 7.19 (s, 4H, *CH*phenyl), 7.02–7.00 (d, *J* = 8.3 Hz, 4H, *o* to *phenyl*OCH_2_- CH_2_), 4.67–4.56 (m, 2H, *CH*CH_3_), 3.75–3.53 (m, 54H, O*CH_2_CH_2_*O), 3.38 (s, 6H, OCH_2_CH_2_*OCH_3_*), 1.36–1.34 (d, *J* = 6.1 Hz, 6H, CHC*H*_3_). Anal. Calcd for C_76_H_102_O_18_: C, 70.02; H, 7.89. Found: C, 69.95; H, 7.93. MALDI-TOF-MS *m*/*z* (M + H)^+^ 1324.

Oligomer **1b**: yield: 40%; ^1^H-NMR (300 MHz, CDCl_3_, δ, ppm): 7.64–7.53 (m, 20H, *o* to CHCH*phenyl*, *m* to CHCH*phenyl*, *o* to *phenyl*OCH_2_CH, *m* to *phenyl*OCH_2_CH, *m* to OCH_2_CH_2_), 7.19 (s, 4H, *CH*phenyl), 7.02–6.99 (d, *J* = 8.8 Hz, 4H, *o* to *phenyl*OCH_2_- CH_2_), 4.66–4.56 (m, 2H, *CH*CH_3_), 3.75–3.54 (m, 107H, O*CH_2_CH_2_*O), 3.38 (s, 6H, OCH_2_CH_2_*OCH_3_*), 1.36–1.34 (d, *J* = 6.2 Hz, 6H, CHC*H*_3_). Anal. Calcd for C_96_H_142_O_28_: C, 66.11; H, 8.21. Found: C, 66.07; H, 8.25. MALDI-TOF-MS *m*/*z* (M)^+^ 1633.

Oligomer **1c**: yield: 40%; ^1^H-NMR (300 MHz, CDCl_3_, δ, ppm): 7.67–7.53 (m, 20H, *o* to CHCH*phenyl*, *m* to CHCH*phenyl*, *o* to *phenyl*OCH_2_CH, *m* to *phenyl*OCH_2_CH, *m* to OCH_2_CH_2_), 7.19 (s, 4H, *CH*phenyl), 7.02–6.99 (d, *J* = 8.8 Hz, 4H, *o* to *phenyl*OCH_2_-CH_2_), 4.65–4.58 (m, 2H, *CH*CH_3_), 3.76–3.55 (m, 170H, O*CH_2_CH_2_*O), 3.38 (s, 6H, OCH_2_CH_2_*OCH_3_*), 1.36–1.34 (d, *J* = 6.2 Hz, 6H, CHC*H*_3_). Anal. Calcd for C_116_H_182_O_38_: C, 63.77; H, 8.40. Found: C, 63.68; H, 8.43. MALDI-TOF-MS *m*/*z* (M)^+^ 1985.

## 4. Conclusions

Coil-rod-coil oligomers **1a**–**1c** consisting of a conjugated rod segment and poly(ethylene oxide) incorporating lateral methyl groups between the rod and coil segment as a coil segment were successfully synthesized. Molecules **1a** self-assembles into hexagonal perforated lamellar, rectangular columnar and oblique columnar nanostructures in the bulk state. However, molecules **1b** and **1c** with longer PEO coil chains than **1a**, self-organize into columnar and hexagonal or tetragonal 3-D nanostructures in the solid state and the liquid crystalline phase. The experimental results reveal that the lateral methyl groups incorporating at the surface of rod and coil segments, dramatically influence the self-assembling behavior in the solid and in the liquid-crystalline mesophase. Moreover, the strategy of incorporating lateral methyl or other alkyl groups into the interface of coil-rod-coil molecular architecture induced construction of three-dimensional functional molecular micelles in the liquid crystalline phase, compared with rod-coil molecules which lack methyl groups.

## Supplementary Information



## Figures and Tables

**Table 1.** Thermal transitions and corresponding enthalpy changes of molecules **1a**–**1c**.

Molecule	Phase transitions (°C) and corresponding enthalpy changes (in brackets) (kJ/mol)	
	Heating	Cooling
**1a**	HPL 106.4 (2.0) Col_rec_ 243.6 (0.9) Col_ob_ 292.4 (0.4) i	i 274.5 (0.8) Col_ob_ 219.7 (1.0) Col_rec_ 104.8 (1.8) HPL
**1b**	Col_rec_ 86.4 (1.9) Col_ob_ 224.6 (0.9) HCP 274.3 (0.4) i	i 249.6 (0.8) HCP 195.9 (1.1) Col_ob_ 80.7 (1.7) Col_rec_
**1c**	Col_hex_ 72.9(1.6) M_tet_ 225.9 (1.4) i	i 205.3 (1.5) M_tet_ 36.4 (0.8) Col_hex_

HPL: hexagonal perforated layer; Col_rec_: rectangular column; Col_ob_: oblique column; Col_hex_: hexagonal column; HCP: hexagonal close packed; M_tet_: body-centered tetragonal micellar; i: isotropic.

**Table 2.** Small-angle X-ray diffraction data for molecule **1a** in the bulk state.

Mesophase (lattice constants)	Reflections/nm		Miller indices (h k l)

	*q_obsd_*	*q_calcd_*	
HPL at 30 °C; *a* = 6.48 nm; *c* = 10.38 nm	1.125	1.125	100
	1.210	1.211	002
	2.037	2.036	2–11
	2.321	2.321	201

Col_rec_ at 160 °C; *a* = 6.49 nm; *b* = 3.43 nm	0.968	0.968	100
	2.064	2.065	110
	2.663	2.662	210
	2.904	2.905	300

Col_ob_ at 230 °C; *a* = 8.61 nm; *b* = 4.66 nm; γ = 135	1.025	1.025	100
	1.908	1.908	010
	2.733	2.734	110
	3.089	3.089	300

*q_obsd_* and *q_calcd_* are the scattering vectors of the observed and calculated reflections; HPL: hexagonal perforated layer; Col_rec_: rectangular column; Col_ob_: oblique column.

**Table 3.** Small-angle X-ray diffraction data for molecule **1b** in the bulk state.

Mesophase (lattice constants)	Reflections/nm		Miller indices (h k l)

	*q_obsd_*	*q_calcd_*	
Col_rec_ at 30 °C *a* = 7.9 nm *b* = 5.2 nm	0.797	0.797	100
	1.453	1.453	110
	1.994	1.994	210
	2.677	2.676	310

Col_ob_ at 160 °C *a* = 9.04 nm *b* = 4.89 nm γ = 135	0.983	0.983	100
	1.822	1.823	010
	1.963	1.965	200
	2.605	2.605	110
	2.945	2.947	300

HCP at 230 °C *a* = 7.75 nm *b* = 15.2 nm	1.025	1.025	101
	1.253	1.253	102
	1. 922	1.922	201
	2.050	2.050	202
	2.833	2.833	301

*q_obsd_* and *q_calcd_* are the scattering vectors of the observed and calculated reflections; HCP: hexagonal closed-packed; Col_rec_: rectangular column; Col_ob_: oblique column.

**Table 4.** Small-angle X-ray diffraction data for molecule **1c** in the bulk state.

Mesophase (lattice constants)	Reflections/nm		Miller indices (h k l)

	*q_obsd_*	*q_calcd_*	
Col_h_ at 30 °C *a* = 7.4 nm	0.983	0.983	100
	1.694	1.694	110
	1.965	1.965	200
	2.590	2.591	210
	2.946	2.947	300

M_tet_ at 160 °C *a* = 9.8 nm *b* = 9.02nm	0.911	0.911	110
	1.396	1.396	002
	1.594	1. 594	211
	1.809	1.808	220
	2.308	2.307	230

*q_obsd_* and *q_calcd_* are the scattering vectors of the observed and calculated reflections; M_tet_: body-centered tetragonal micelle; Col_h_: hexagonal column.
