# Supplementary material for: Liquid Crystalline Assembly of Coil-Rod-Coil Molecules with Lateral Methyl Groups into 3-D Hexagonal and Tetragonal Assemblies
Source: Int J Mol Sci. 2014 Apr 2;15(4):5634–48. doi: 10.3390/ijms15045634 (PMC4013586; doi:10.3390/ijms15045634)
Supplement: Supplementary file 1 [file ijms-15-05634-s001.pdf]

Supplementary Information

S1. Experimental Section

S1.1. Synthesis of the Coil Segment

Figure S1. Synthetic route of Coil Segment **3**.

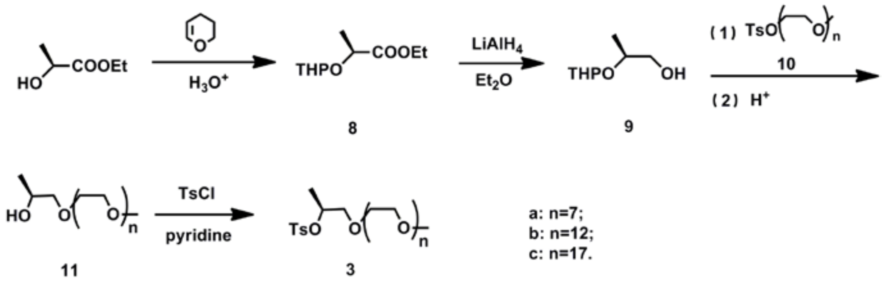

S1.1.1. Synthesis of Compounds **8**

To a solution of Ethyl L-(−)-lactate (36.5 g, 0.308 mol), and 3,4-dihydro-2H-pyran (42.1 g, 0.5 mol) were slowly added 10 drops of concentrated HCl at 0 °C. The solution was stirred for 10 h while it reached room temperature. Na_2CO_3 (3 g) was added, and stirring was continued for 10 h. The reaction mixture was then filtered, concentrated to small volume, and distilled to give **8** (51.9 g, yield 83%).

S1.1.2. Synthesis of Compounds **9**

To a suspension of LiAlH (12.0 g, 0.316 mol) in dry diethyl ether (80 mL) was added a solution of **15** (51.9 g, 0.257 mol) in diethyl ether (40 mL) at 0 °C under nitrogen atmosphere over 8 h. After 12 h at room temperature, the reaction mixture was refluxed for 5 h. Methyl acetate (5 g), 10% NaOH (20 mL), and water (80 mL) were then added in that order to the mixture cooled at room temperature. The precipitate was filtered off, and the solution was concentrated, dried over K_2CO_3 and finally distilled to give **9** (35.2 g, yield 86%).

S1.1.3. Synthesis of Compounds **11a**–**11c**

These compounds were synthesized according to the same procedure; a representative example is described for **11a**. To a 250 mL two-neck flask compound **9** (5 g, 31.2 mmol), dry THF (100 mL) was added. After stirring for several min at 0 °C, NaH (3.37 g, 84 mmol) was added slowly. And then **10a** (10.98 g, 15.6 mmol) was added dropwise for 1.5 h at 0 °C. The mixture was refluxed for 24 h. The reaction was concentrated by evaporation and extracted with dichloromethane. The combined organic portions were dried over $MgSO_4$ and filtered. The residue was purified by flash column chromatography with EA/CH_3OH = 4/1 as eluent to give product **11a** (9 g, 83%) as a colourless liquid.

S1.1.4. Synthesis of Compounds **3a**–**3c**

These compounds were synthesized according to the same procedure, a representative example is described for **3a**. To a 250 mL round bottom flask **11a** (4 g, 6.46 mmol), *p*-Toluenesulfonyl chloride (2.46 g, 12.9 mmol), pyridine (20 mL), and dry Dichloromethane (100 mL) were added. After being

stirred at room temperature for 18 h, 30 mL water was added into the reaction mixture, the aqueous phase was slowly acidified with cold 3 N HC1 to pH = 2–3 and extracted with dichloromethane (3 × 50 mL). Then the organic fractions were dried over $MgSO_4$. The residue was purified by flash column chromatography with EA/CH_3OH = 10/1(*v*/*v*) as eluent to give product **3a** (3.5 g, 70%) as colourless liquid.

Figure S2. ^{1}H-NMR spectra of molecules **1a** in $CDCl_3$.

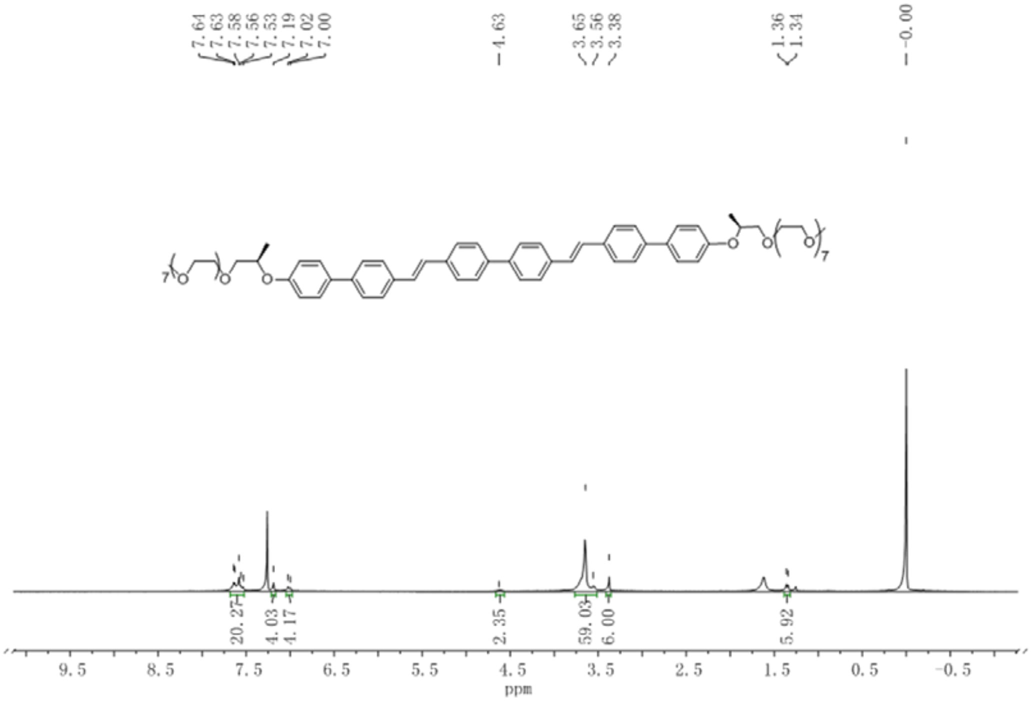

Figure S3. MALDI-TOF-Mass spectrum of **1a**, **1b**, **1c** (matrix: CHCA).

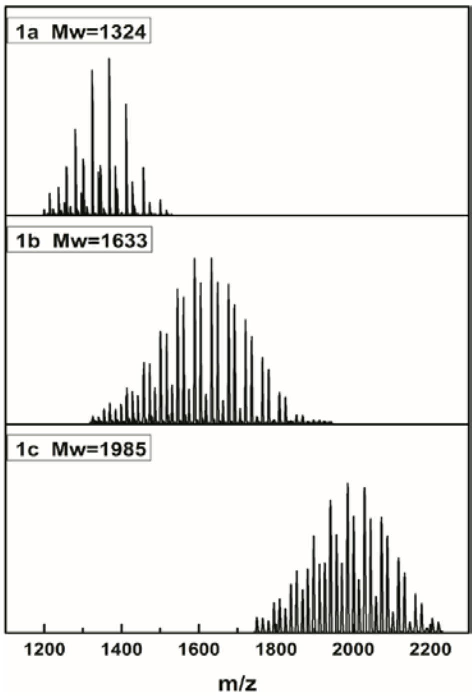

Figure S4. ^{1}H-NMR spectra of molecules **1b** in $CDCl_3$.

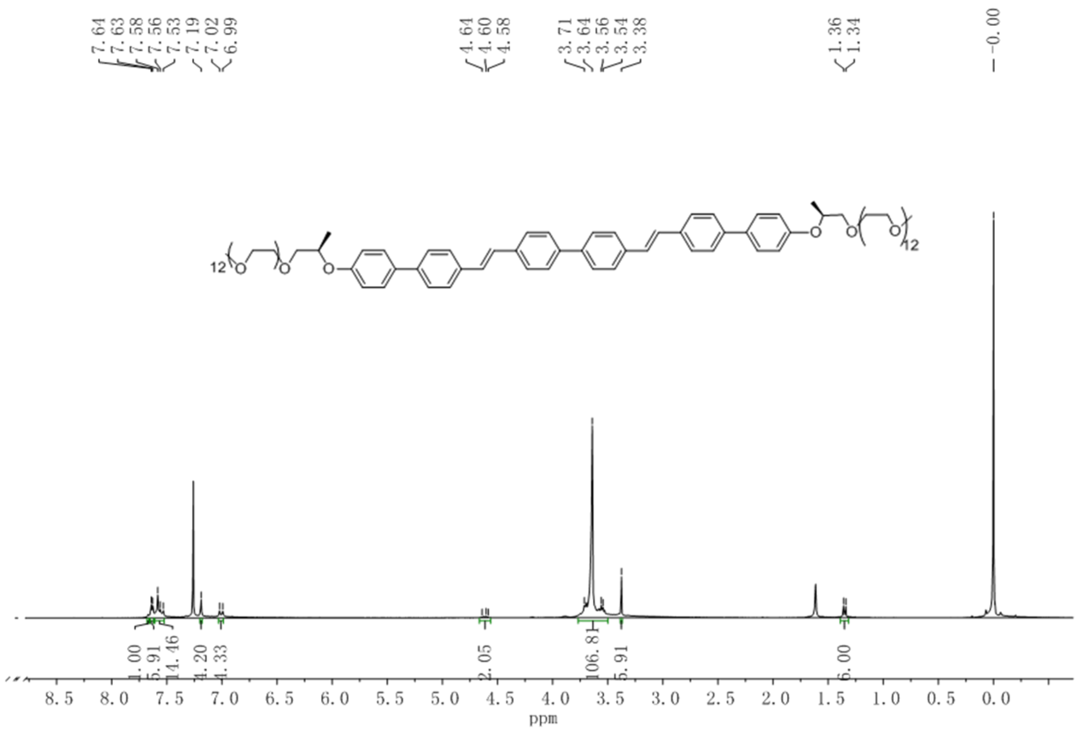

Figure S5. ^{1}H-NMR spectra of molecules **1c** in $CDCl_3$.

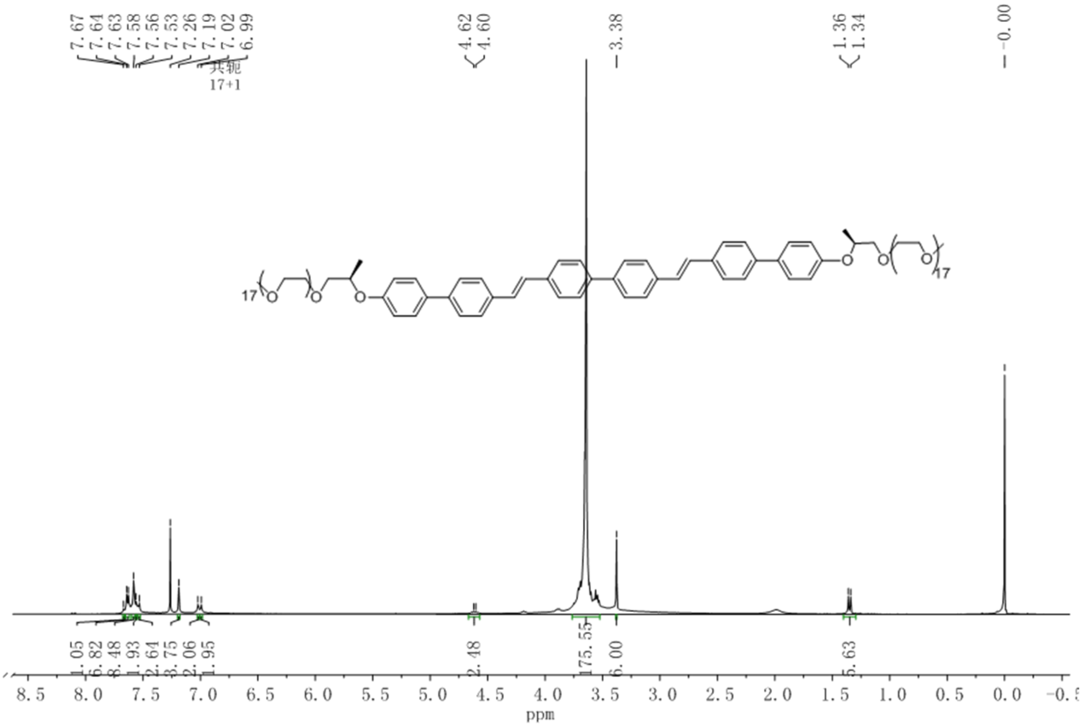

S2. Results and Discussion

Figure S6. DSC traces (10 °C/min) recorded during the second heating scan and the first cooling scan of the series of molecules (**a**) **1a**; (**b**) **1b**; (**c**) **1c**.

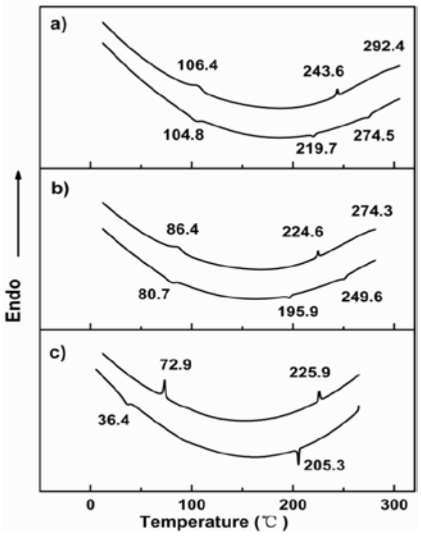

Table S1. Thermal transitions and corresponding enthalpy changes of **2b** and **2c**.

**Molecule**	**Phase transitions (°C) and corresponding enthalpy changes (in brackets) (kJ/mol)**	
	**Heating**	**Cooling**
**2b**	HPL 98.5 (2.0) Col_r 236.4 (0.9) Col_{ob} 288.5 (0.4) i	i 267.3 (0.8) Col_{ob} 212.6 (1.0) Col_r 98.1 (1.8) HPL
**2c**	HPL 92.1 (1.6) Col_r 235.4 (1.4) i	f

HPL: hexagonal perforated layer; Col_r: rectangular column; Col_{ob}: oblique column; i: isotropic.

It is obvious that increasing the flexible chain length or incorporating side groups at the surface of rod and coil domains lead to reduce phase transition temperatures. This trend is believed to be attributed to a reducion in the extent of packing constraints of rod segments as a result of increasing the flexible chain length or incorporating side groups, subsequently reducing the driving force of pi-pi stacking.

Figure S7. Representative optical polarized micrograph (×100) of the texture exhibited by (**a**) hexagonal column structure of **1c** in the crystal phase; (**b**) Body-centered tetragonal structure of **1c** at the transition from the anisotropic liquid crystal phase.

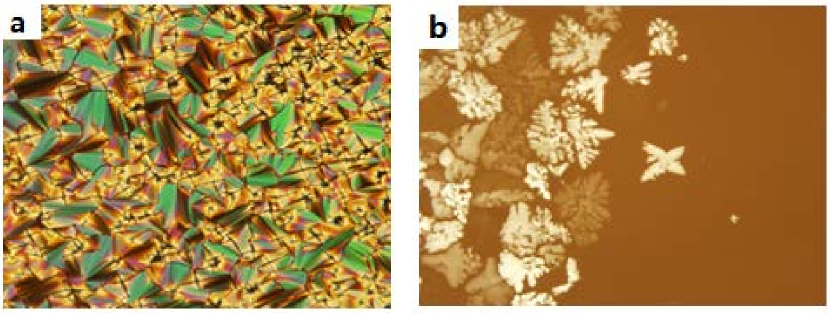

*Aggregation Behavior of Molecule **1a–1c** in Aqueous Solution*

Molecules **1a**–**1c** can be considered as a novel class of amphiphiles due to the fact that these molecules consist of hydrophilic poly(ethylene oxide) flexible chains and hydrophobic conjugated rod segment. Aggregation behavior of molecules **1a**–**1c** was subsequently studied in aqueous solution using UV–vis and fluorescence spectra. The absorption spectra of **1a**–**1c** in aqueous solution (0.02 wt %) exhibit a blue-shifted absorption maxima compared to those of chloroform solutions. This is maybe caused by the cooperative effect between the hydrophilic and hydrophobic interactions and the π–π stacking interactions between the aromatic segments. The fluorescence spectra of **1a**–**1c** in chloroform solution (0.02 wt %) exhibit a strong emission maximum at 430 and 455 nm respectively. However, the emission maximum in aqueous solution is significantly quenched, strongly indicative of aggregation of the conjugated rod segments.

Figure S8. (**a**) Absorption and emission spectra of **1a** in chloroform and aqueous solution (0.002 wt %); (**b**) Absorption and emission spectra of **1b** in chloroform and aqueous solution (0.002 wt %); (**c**) Absorption and emission spectra of **1c** in chloroform and aqueous solution (0.002 wt %).

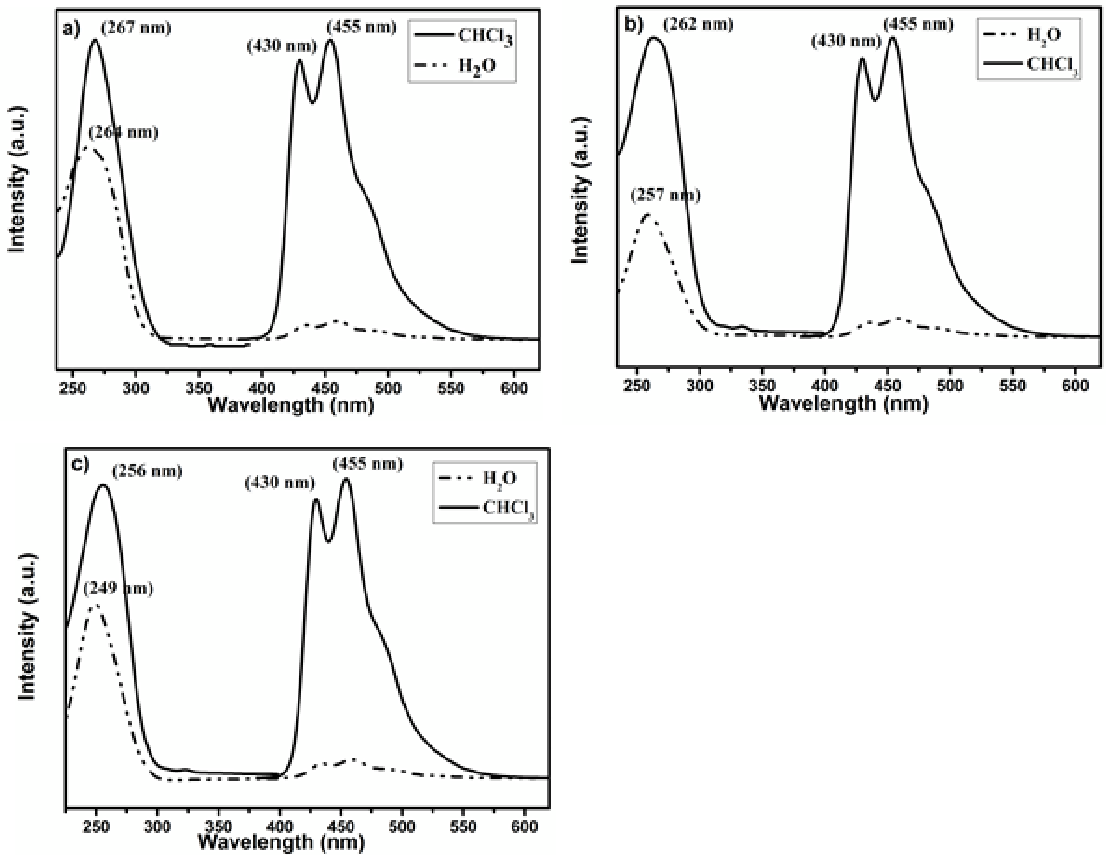

© 2014 by the authors; licensee MDPI, Basel, Switzerland. This article is an open access article distributed under the terms and conditions of the Creative Commons Attribution license (http://creativecommons.org/licenses/by/3.0/).
